# Interferon Signature in the Blood in Inflammatory Common Variable Immune Deficiency

**DOI:** 10.1371/journal.pone.0074893

**Published:** 2013-09-17

**Authors:** Joon Park, Indira Munagala, Hui Xu, Derek Blankenship, Patrick Maffucci, Damien Chaussabel, Jacques Banchereau, Virginia Pascual, Charlotte Cunningham-Rundles

**Affiliations:** 1 Department of Medicine and the Immunology Institute, Icahn School of Medicine at Mount Sinai, New York, New York, United States of America; 2 Baylor Institute for Immunology Research, Dallas, Texas, United States of America; 3 Systems Immunology, Benaroya Research Institute, Seattle, Washington, United States of America; 4 Baylor HealthCare System, Institute for Health Care Research and Improvement, Department of Quantitative Sciences, Dallas, Texas, United States of America; Cordelier Research Center, INSERMU872-Team16, France

## Abstract

About half of all subjects with common variable immune deficiency (CVID) are afflicted with inflammatory complications including hematologic autoimmunity, granulomatous infiltrations, interstitial lung disease, lymphoid hyperplasia and/or gastrointestinal inflammatory disease. The pathogenesis of these conditions is poorly understood but singly and in aggregate, these lead to significantly increased (11 fold) morbidity and mortality, not experienced by CVID subjects without these complications. To explore the dysregulated networks in these subjects, we applied whole blood transcriptional profiling to 91 CVID subjects, 47 with inflammatory conditions and 44 without, in comparison to subjects with XLA and healthy controls. As compared to other CVID subjects, males with XLA or healthy controls, the signature of CVID subjects with inflammatory complications was distinguished by a marked up-regulation of IFN responsive genes. Chronic up-regulation of IFN pathways is known to occur in autoimmune disease due to activation of TLRs and other still unclarified cytoplasmic sensors. As subjects with inflammatory complications were also more likely to be lymphopenic, have reduced B cell numbers, and a greater reduction of B, T and plasma cell networks, we suggest that more impaired adaptive immunity in these subjects may lead to chronic activation of innate IFN pathways in response to environmental antigens. The unbiased use of whole blood transcriptome analysis may provides a tool for distinguishing CVID subjects who are at risk for increased morbidity and earlier mortality. As more effective therapeutic options are developed, whole blood transcriptome analyses could also provide an efficient means of monitoring the effects of treatment of the inflammatory phenotype.

## Introduction

Common variable immunodeficiency (CVID) is a relative common primary immune deficiency characterized by low levels of serum immunoglobulin G, A, and/or M, coupled with a lack of production of specific IgG antibodies [1] [2,3]. While mutations autosomal genes leading to loss of B cell function have been identified in a few rare cases [4], [5,6,7][8,9],, for the great majority of patients, the genetic basis remains unknown. As for other predominantly B cell defects, immunoglobulin (Ig) replacement is the mainstay of treatment, and while this therapy effectively reduces the incidence of bacterial infections, it does not prevent or ameliorate the non-infectious organ-damaging complications which develop in about half of all subjects [10,11]. These complications include hematologic and organ-specific autoimmunity, granulomatous infiltrations, interstitial lung disease, lymphoid hyperplasia, gastrointestinal inflammatory disease, cancer, and lymphoma [12,13,14,15,16]. As bacterial infections have become less common, these inflammatory conditions, shown to lead to significant increased morbidity and earlier mortality, are now one of the most important avenues of investigation in CVID. An analysis of our CVID cohort of 473 patients followed over 4 decades showed that subjects with suffering from inflammatory conditions had an 11-fold increase in mortality as compared to those without [10]. These observations parallel data compiled on a large cohort of European CVID subjects, demonstrating that CVID subjects have remarkably stable clinical phenotypes over decades of follow-up, that are closely related to long-term outcomes [15]. Unfortunately, the existing biomarkers do not allow us to identify those CVID subjects that will more likely develop inflammatory complications, and the best therapeutic measures to treat these patients, have remained undefined.

Previous whole blood transcriptional signatures based on RNA microarray analyses have demonstrated specific pathways activated in autoimmune, infectious and neoplastic diseases [17,18,19,20,21]. This systems biology approach has improved diagnosis and provided better understanding of disease pathogenesis by revealing unique signatures and in some cases, a useful pharmacologic approach. For example, the application of microarray blood transcriptional profiles led to a better understanding of systemic onset juvenile arthritis, and the identification of IL-1as an important therapeutic target [22,23]. Here we applied blood transcriptional profiling to characterize the immunologic networks in subjects with CVID who have the inflammatory complications to identify clues as to pathogenesis and potentially better modes of treatment. Blood of CVID subjects with inflammatory complications demonstrated a marked up regulation of IFN responsive genes.

## Materials and Methods

### Patient population

CVID subjects fulfilled the standard diagnostic criteria, including significantly decreased levels of serum IgG, IgA, and/or IgM and poor or absent specific antibody production [1,24]. Subjects with or without a set of characteristic inflammatory complications, as previously designated, were enrolled. These included hematologic or organ-specific autoimmunity, biopsy-proven granulomatous disease, interstitial lung disease leading to impaired lung functions, lymphoid hyperplasia with splenomegaly or gastrointestinal inflammatory disease [10,15,24]. All subjects were free from inter-current infections, and were not taking antibiotics or any immune-modifying medications at the time of study. Blood was taken before the interval intravenous immune globulin (Ig) infusions, or between subcutaneous Ig administrations. The training set included 59 patients (29 females, 30 males) age 11 through 88 (mean age, 44.7 years) and 21 healthy adult volunteers. The test set included 32 CVID patients, 16 males and females (age 11 to 69, mean age, 42 years), and 15 control samples. For clinical and immunologic data, baseline laboratory results before initiating Ig replacement were used; other test results were those done at the time of diagnosis or the first clinic visit (Table 1). Six patients with X-linked agammaglobulinemia (XLA) were included in the test set as controls as they also have loss of antibody production and are treated with periodic Ig replacement. For 8 of the CVID subjects, blood samples were also taken both before and 5 or 6 days after IVIg infusions to determine if Ig administration altered mRNA signatures. The study was approved by the Mount Sinai Medical Center Institutional Review Board and written informed consent was obtained from each patient or parent.

**Table 1:** Subjects in Training and Test Sets.

**Variable**	**Training Sets (N=59**)	**Test Set (N=32**)	**P-value**
Age at time of analysis, Median (Years, Min, Max)	43 (10-88)	42.0 (12-69)	0.42
SEX, No. (%)			
Female	29 (49%)	16 (50%)	1.00
Male	30 (51%)	16 (50%)	1.00
B+CD27+IgM-IgD-, Median (Min, Max)	1.0 (0.0, 22.4)	1.1 (0.0, 6.0)	0.90
IgG, Median (Min, Max)	246.5 (6.0, 898.0)	187.0 (13.0, 597.0)	0.60
IgA, Median (Min, Max)	8.2 (0.0, 535.0)	7.0 (0.0, 95.0)	0.21
IgM, Median (Min, Max)	20.0 (3.0, 205.0)	19.0 (1.0, 247.0)	0.88
Complication, No. (%)			0.66
No	30 (51%)	14 (44%)	
Yes	29 (49%)	18 (56%)	
Autoimmunity+, No. (%)			0.63
No	41 (69%)	24 (75%)	
Yes	18 (31%)	8 (25%)	
Splenomegaly+, No. (%)			0.80
No	44 (75%)	25 (78%)	
Yes	15 (25%)	7 (22%)	
Granuloma+ (Biopsy proven), No. (%)			0.77
No	50 (85%)	26 (81%)	
Yes	9 (15%)	6 (19%)	
Lymphadenopathy/splenomegaly+, No. (%)			0.43
No	44 (75%)	27 (84%)	
Yes	15 (25%)	5 (16%)	
Enteropathy, No. (%)			0.10
No	54 (92%)	25 (78%)	
Yes	5 (8%)	7 (22%)	

+ subjects commonly had more than one of these conditions.

### RNA sampling and processing for microarray analysis

Blood samples were obtained in Tempus tubes (Applied Biosystems) vigorously mixed and stored at -80°C. Total RNA was isolated from the whole blood lysate using the MagMAX RNA Isolation Kit for Blood RNA Tubes (Ambion/ Life technologies). RNA integrity was assessed using an Agilent 2100 Bioanalyser (Agilent Technologies) and Caliper (PerkinElmer). Isolated total RNA was globin-reduced using the GLOBINclear 96-well format kit (Ambion/Life technologies). Globin-reduced RNA was amplified and labeled using the Illumina TotalPrep RNA Amplification Kit (Ambion). Labeled cRNA was hybridized overnight to Illumina Human HT-12 V3 BeadChip (training) and HT-12 V4 BeadChip (test set) arrays (Illumina), which contained more than 48,000 probes in HT-12 V3 BeadChip and 47,231 probes in HT-12 V4 BeadChip. The assays were then washed, blocked, stained, and scanned on an Illumina BeadStation 500 (training and test sets) and iScan Control Software v 3.3.28 (test set). For the training set, Illumina BeadStudio version 2 software was used to generate signal intensity values from the scans [18,25,26]. For the test set, Genome Studio v2011.1 was used to generate signal intensity values.

### Microarray Data Processing

The microarray background was subtracted and the average signal intensity for each sample was scaled to the global average signal intensity for all samples, across multiple arrays and chips. For the purpose of comparability between the training and test data sets, all analyses were applied on the common probes from HT12V3 and V4 chips. Expression values less than 10 were set to 10 and log (base 2) transformed. Microarray data were filtered by using a present call (detection p-value less than 0.01 in at least one sample) and standard deviation filtering criteria (standard deviation of expression values across samples greater than the median of those standard deviations). Batch effect correction was applied using the group batch profiling technique available in JMP/Genomics (SAS Institute Inc., NC) for all data sets. Per-gene normalization was applied for displaying results in heat maps by dividing each probe intensity by the median intensity value for all samples or the healthy controls where specified.

### Statistical Data Methods

For microarray data, unsupervised analysis was performed to create an unbiased grouping of samples on the basis of their molecular profiles, independently of disease, immunologic phenotype or clinical classification. Transcripts meeting the filtering criteria and further downstream analysis were then subjected to hierarchical clustering using GeneSpring. Hierarchical clustering is an iteratively agglomerative clustering method performed to find similar transcriptional expression patterns and to produce gene trees or condition trees representing those similarities. The hierarchical clustering performed in our datasets was calculated through the average linkage while the similarity or dissimilarity of gene expression profiles was measured using Pearson correlation. The color conventions for both heat maps include red for over-expressed transcripts, blue for under-expressed transcripts, and yellow transcripts that do not deviate from the median. Microarray data were further analyzed using analysis of variance (ANOVA) to identify differentially expressed probes in the comparisons among patients with complications, patients without complications, and healthy controls. Multiple testing corrections were applied using a false discovery rate (FDR) of 0.05 [27] or a Bonferroni correction with an alpha of 0.05.

Transcriptional modular analysis was also performed as described previously [25,26]. Briefly, this mining strategy relies on whole genome profiles obtained from sets of patients with a wide range of immune-mediated diseases for the construction of a large co-clustering network. Highly connected sub-networks, also called modules, are extracted and the resulting gene sets used as a framework to simplify downstream analysis and interpretation of individual datasets. Here a framework constituted of 260 modules spanning nearly 15,000 individual probes was used. The number and proportion of differentially expressed probes was determined by module for each comparison. Modules containing a proportion of significantly up- or down-regulated probes greater than 10% were considered up- or down-regulated, respectively. The results of the training set were then validated in separate test set. Demographic and clinical parameters for the CVID subjects in test and training sets were compared using the Fisher’s exact test for categorical variables and the Mann-Whitney test for numeric variables. Statistical analyses of microarray data and clinical data were performed using Genespring v7.3 JMP/Genomics (v5.0) and SAS software (v9.2) (both from SAS Institute Inc., NC). Microarray data is available at the NCBI GENE Expression Omnibus (GEO), web site http://www.ncbi.nlm.nih.gov/geo/info/geo_illu.html.

### Production of interferons

To examine T cell production of IFNγ, PBMCs were isolated from peripheral blood samples of patients and controls by Ficoll-Paque (GE Healthcare, Uppsala, Sweden) and 5x10^5^ cells stimulated with 1.25µL/mL and 2µL/mL CD3/CD28 Dynabeads beads (Invitrogen), or 2µg/mL PHA in 96-well plates. Supernatants were harvested after 72 hours, diluted (1:10, 1:50, and 1:100) and assessed for IFNγ using OptEIA human IFNγ ELISA kit (BD Biosciences). For IFN-α production, control and CVID PBMCs at 2 × 10^5^/mL were stimulated for 48 hours with 100, 500 or 1000 µmol/L 7-allyl-7,8-dihydro-8-oxo-guanosine (loxoribine; TLR7 agonist) and 0.25 and 0.5 as an imidazoquinoline TLR7/8 agonist (InvivoGen) as previously described [28], IFN-α levels in harvested supernatants were assessed by ELISA (Bender Medsystems, Burlingame, CA).

## Results

### Transcriptional signatures in CVID

**Figure 1:**
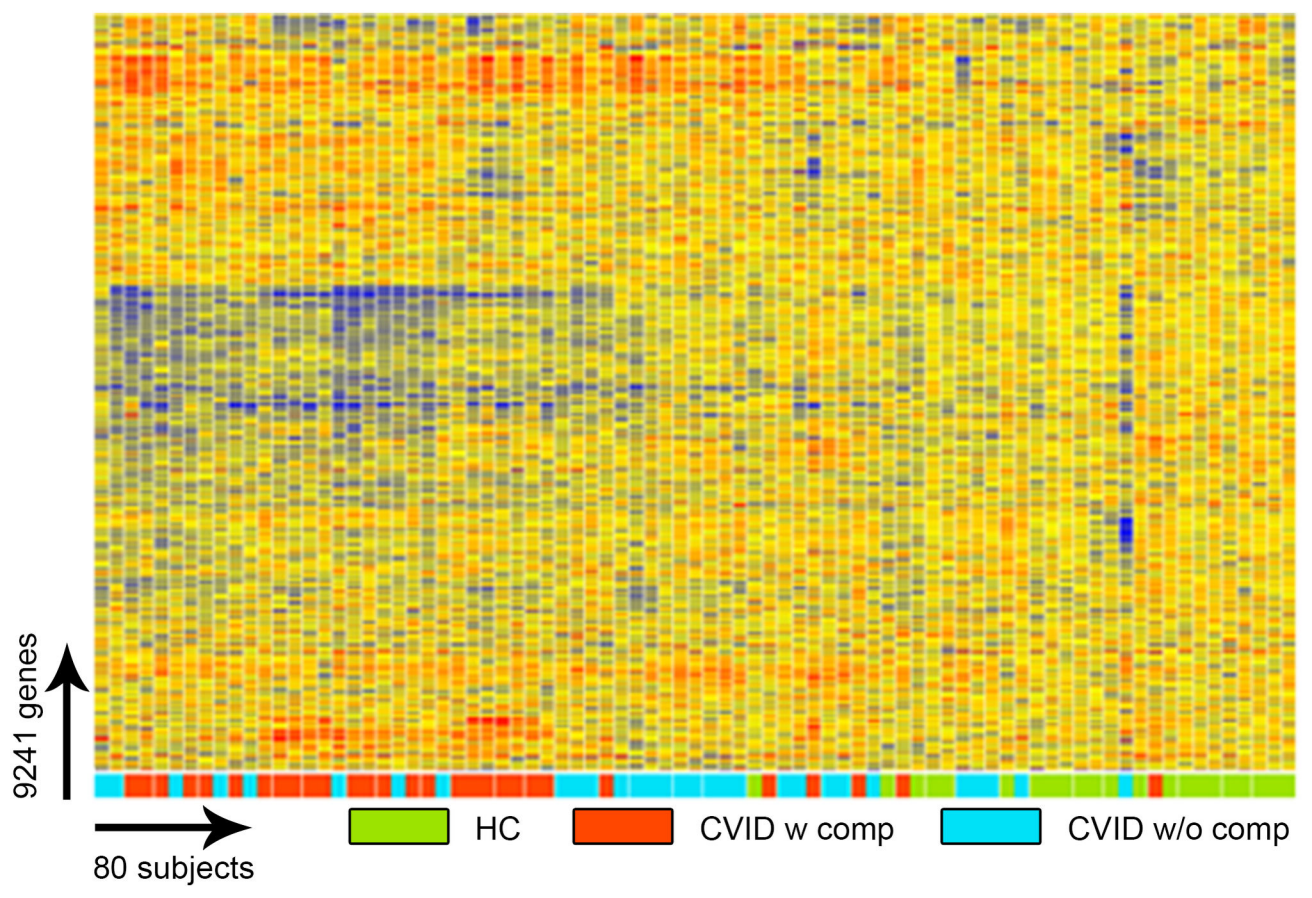
Gene expression analysis of CVID blood (training set) compared to healthy controls. Unsupervised clustering of 9,241 RNA transcripts expressed in whole blood cells selected by Present At least Once (PALO), differentiates CVID patients from healthy controls in the training set.

**Figure 2:**
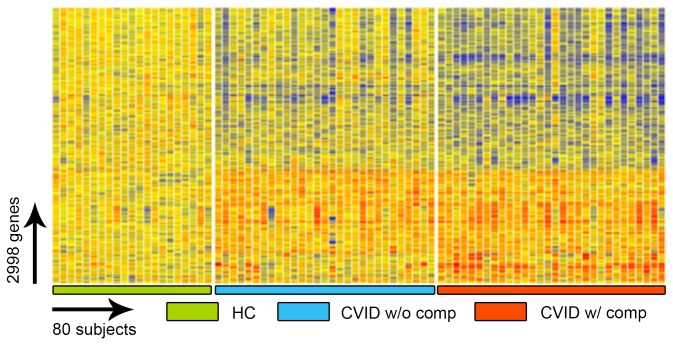
ANOVA test comparing CVID blood (training set) to healthy controls. The ANOVA test (FDR 0.05) identifies 2,998 transcripts differentially expressed in whole blood cells of CVID patients with and without inflammatory complications and healthy controls.

**Figure 3:**
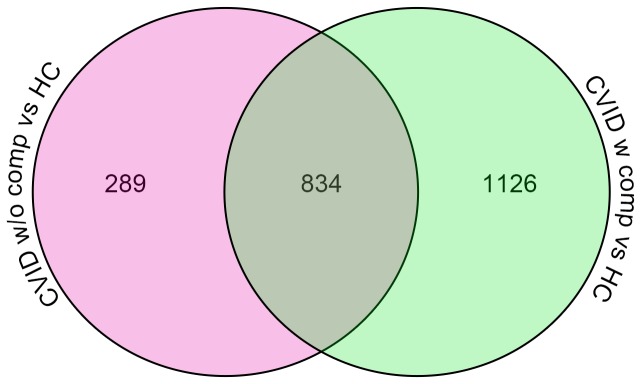
Venn diagram representing the number of common and unique transcripts differentially expressed in patients, compared to healthy controls. Blood from subjects with inflammatory complications contained more upregulated transcripts as compared to those without complications.

To identify potential transcriptional signatures characteristic of CVID subjects with inflammatory conditions, 59 subjects and 15 controls were examined in a training set, and 32 other CVID subjects and 16 controls in a test set. Each group of CVID subjects contained males and females of similar age and sex, with similar baseline serum immune globulin levels, and numbers of subjects with hematologic or organ specific autoimmunity, granulomatous disease, interstitial lung disease, lymphoid hyperplasia/splenomegaly and/or gastrointestinal inflammatory disease (Table 1). Subjects with and without inflammatory complications had similar immunologic profiles with the exception of significantly reduced numbers of lymphocytes, fewer B cells and fewer isotype switched memory B cells for those with complications, as previously noted for such subjects [29,30] (Table 2). The training set was used to identify the profile of CVID subjects with and without inflammatory complications in comparison to controls; the test set confirmed the differential gene expression profiles. The heat map shown in Figure 1 represents the unsupervised hierarchically clustered profile of 9,241 RNA transcripts selected by Present At least Once (PALO) from blood of 59 CVID subjects in the training set, as normalized to 24 control samples, all vertically aligned. Notable were the marked differences in up- and down-regulated transcriptional patterns, largely separating CVID subjects from controls, and demonstrating differential signatures for the CVID subjects with inflammatory complications. Hierarchical clustering according to transcripts that passed the multiple test correction (ANOVA, FDR 0.05) showed that the 29 CVID subjects with inflammatory conditions displayed many more differentially expressed transcripts compared to the 30 CVID subjects without these complications (Figure 2) although some overlap was noted between populations. Thus, the CVID group with complications contributed most of the transcriptional differences observed between CVID and healthy subjects (Figure 3).

**Table 2:** Comparing CVID subjects with and without Inflammatory Complications.

	**Complications (25^th^-75^th^ percentile**)** n=47**	**No Complications (25^th^-75^th^ percentile**)** n=44**	*P-value
Age	42 yrs (32-49.5)	43 yrs (38.8-55.8)	0.36
IgG	207.5 mg/dl (94.3-341.5)	202 mg/dl (67.5-352.8)	0.75
IgA	7 mg/dl (0-15.5)	8 mg/dl (6.0-20.5)	0.12
IgM	18 mg/dl (6.5-46.0)	22 mg/dl (12-40)	0.99
B cell %	7% (0.2-14.5)	9.5% (0.1-13.9)	0.001
Isotype switched memory B cells	0.65% (0-1.6)	1.3% (0.45-2.4)	0.001
Absolute Lymphocyte	1300 (1100-3100)	1100 (800-1600)	0.03
T cells	75% (65.3-82.5)	75% (65.3-82.5)	0.63
CD4+ T cells	581 (513-816)	579 (336-708)	0.5

* Mann-Whitney 2-tailed test.

**Figure 4:**
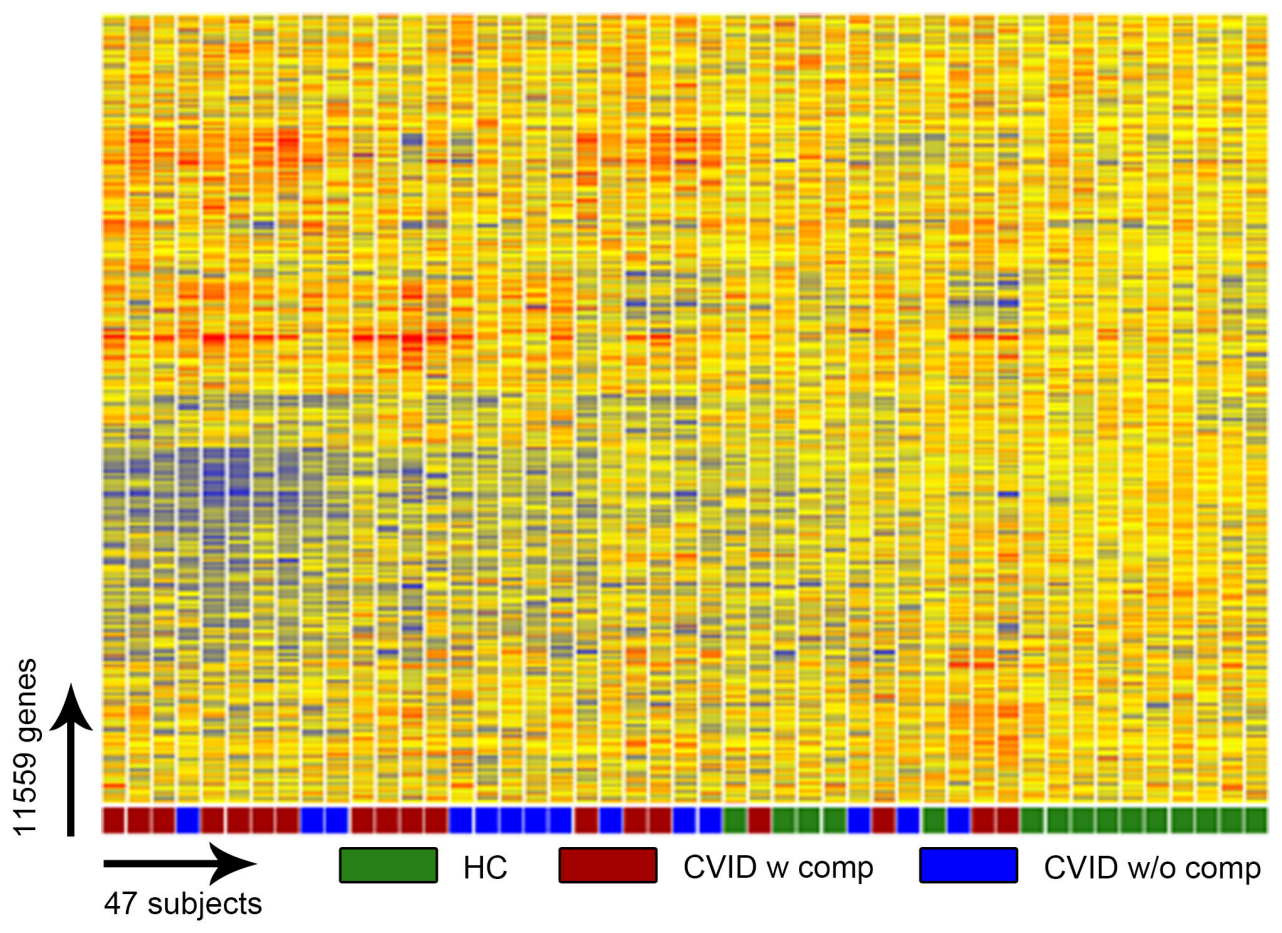
Gene expression analysis of CVID blood (test set) compared to healthy controls. Unsupervised clustering of 11,559 RNA transcripts in whole blood cells, selected by Present At least Once (PALO) differentiates CVID patients from healthy controls in the test set.

**Figure 5:**
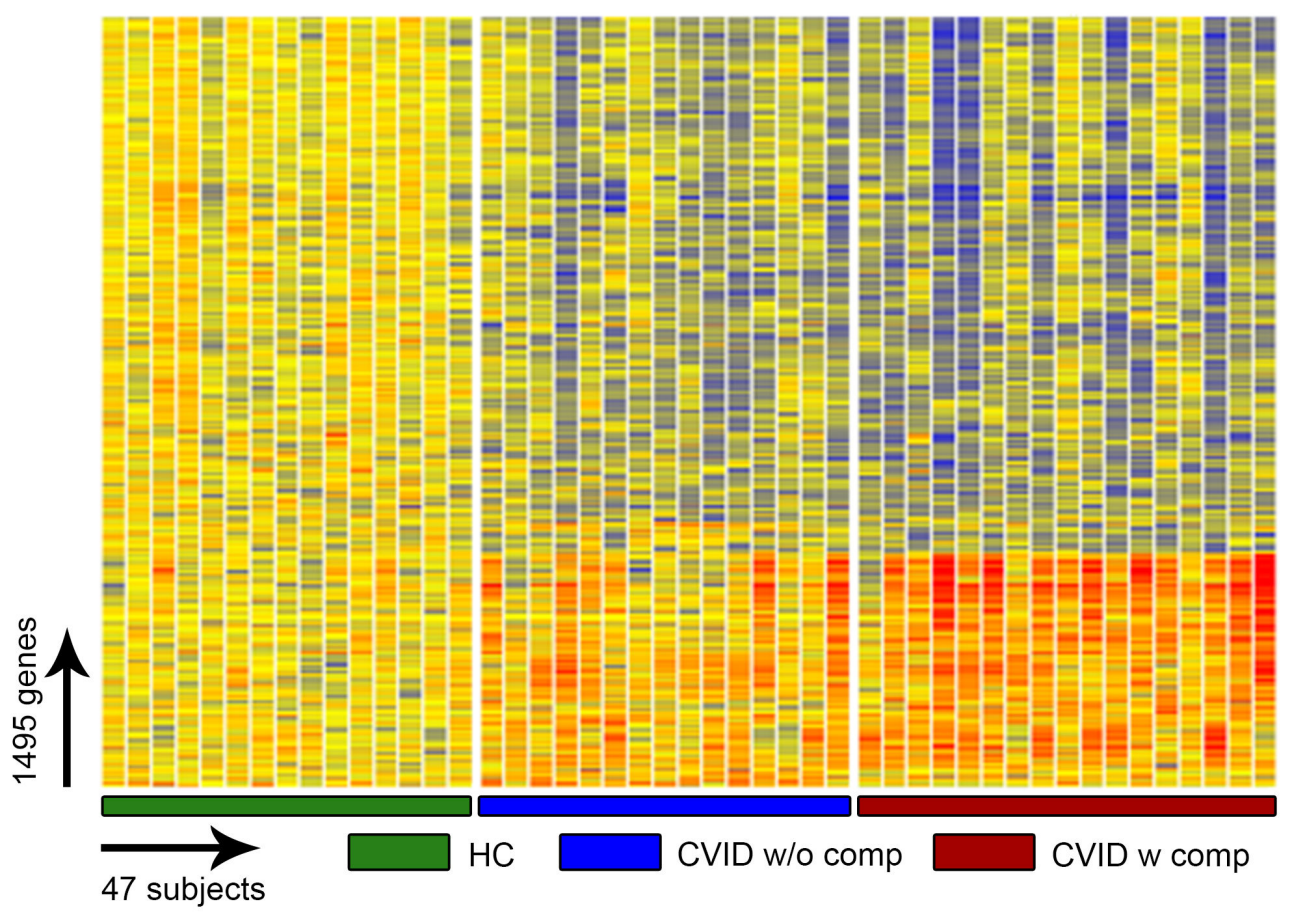
ANOVA test (FDR 0.05) comparing CVID blood (test set) healthy controls. This test identified 1495 transcripts differentially expressed in the blood of CVID patients, identified here with and without inflammatory complications and healthy controls.

**Figure 6:**
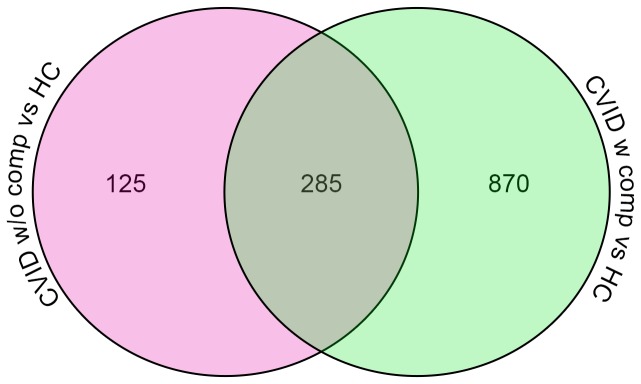
Venn diagram representing the number of common and unique transcripts. Whole blood of subjects with inflammatory complications displayed differentially expressed transcripts as compared to those without such complications or healthy controls.

To validate the emerging transcriptional profiles, we recruited an additional 32 CVID subjects, 18 with and 14 without inflammatory conditions and 15 additional controls. Unsupervised clustering of all transcripts present in the blood of these subjects again distinguished the majority of CVID subjects from healthy controls (Figure 4), and as described for the training cohort, a significant divergence of the signature of CVID subjects with inflammatory complications (Figure 5) revealing a large number of transcriptional differences for these CVID subjects as compared both controls and other CVID subjects (Figure 6).

### Transcriptional modules

**Figure 7:**
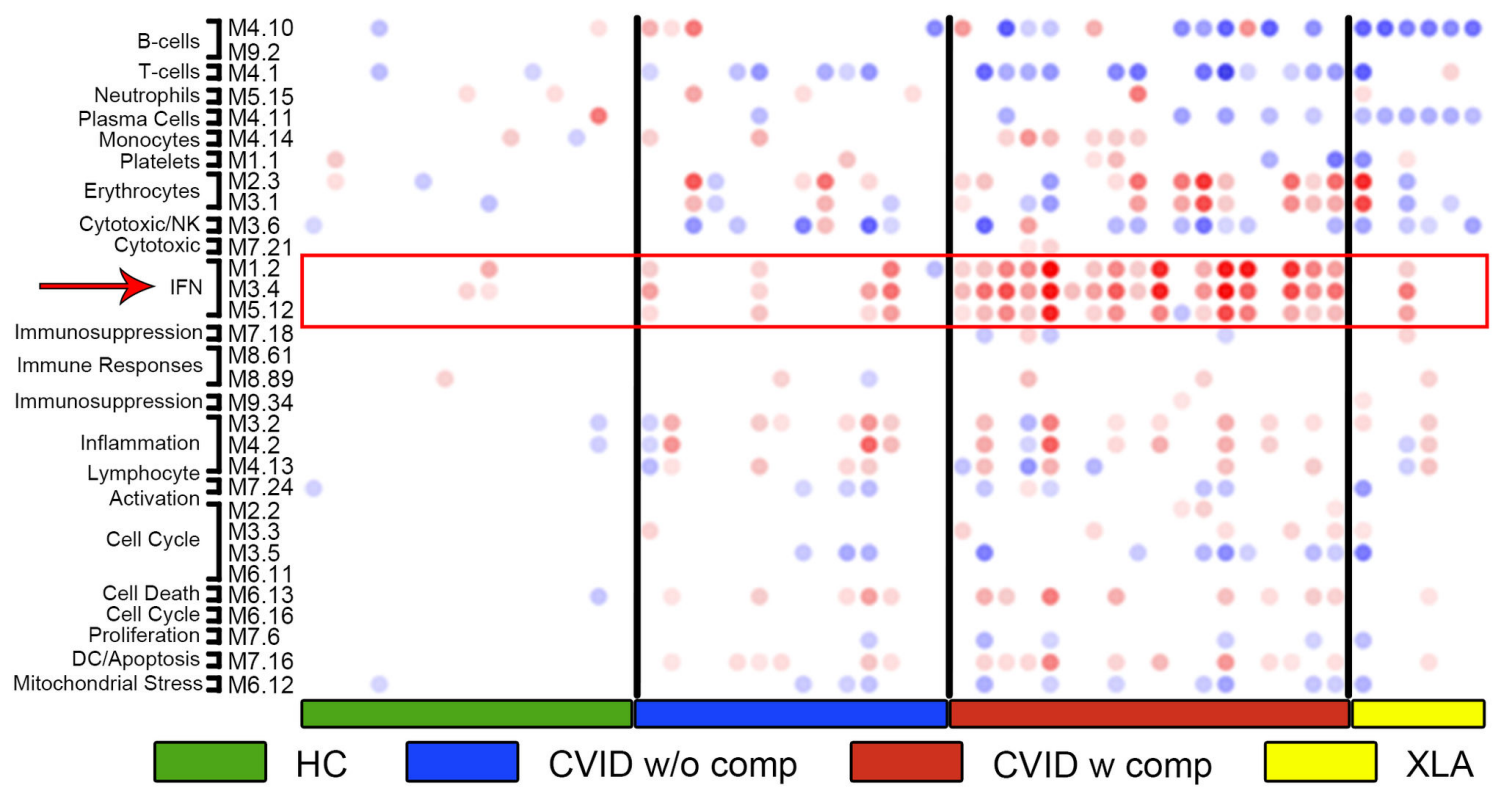
Modular analysis of differentially expressed transcripts in CVID. Whole blood modular signatures of CVID subjects in the test set identified significant down regulation of B-cell and T-cell modules and up-regulation of IFN-related modules, preferentially in CVID patients with inflammatory complications. Columns represent the profiles on individual patients and controls, healthy controls and XLA subjects. Spot intensity (red = increased, blue = decreased) indicates transcript abundance within each module as noted.

**Figure 8:**
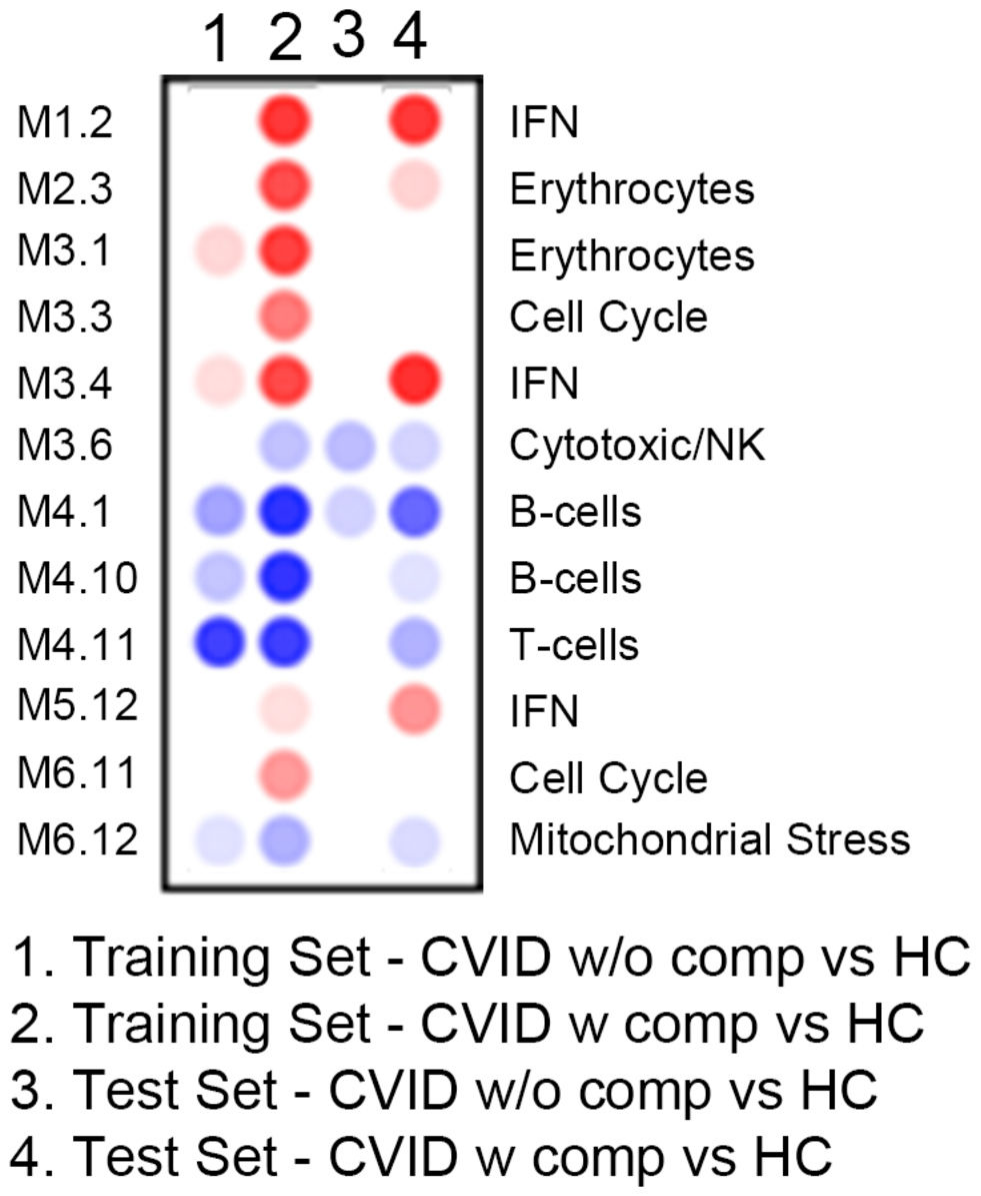
Statistically significant modules from both training and test sets compared to healthy controls. Columns are as labeled for subjects with or without complications vs. healthy controls (HC).

To gain insight into potential biological pathways responsible for the transcriptional alterations observed in CVID blood, we then applied a pre-established modular analysis framework that groups blood genes according to shared expression patterns across health and disease [26]. Using this approach, control samples showed negligible perturbation in modular expression while samples from CVID subjects showed clear-cut modular differences (Figure 7). Subjects with XLA were also included in the test set as controls, as these subjects have loss of B cell function but very rarely the inflammatory complications found in CVID. Modular analyses showed that subjects with CVID with one or more of the inflammatory/autoimmune conditions displayed significantly over-expressed interferon-related modules (M1.2 M3.4 and M5.12), and a pronounced down-regulation of transcripts related to the B cell, plasma cell, and T cell modules (M4.10, M9.2, and M4.11, M4.1 respectively) as compared to CVID subjects without these conditions. As expected, the XLA samples showed significantly down-regulated B cell and plasma cell modules, however, other transcripts/modules dysregulated in subjects with CVID, including T cell-related and IFN-related genes, were for the most part not changed in these XLA subjects. Transcripts reflective of neutrophil activation were not increased (M5.15) in these subjects as compared to controls, although these were found (and have been annotated) in other work using this platform [19,31]. Comparing by modular analyses from both training and test sets to that of healthy controls, and the CVID groups to each other, showed that the IFN modules were the most up-regulated in patients with inflammatory conditions while B and T cell-related modules were the most down regulated across all CVID patients (Figure 8). For both training and tests, we attempted to segregate CVID subjects with autoimmunity, granulomatous disease, enteropathy and/or splenomegaly/lymphoid hypertrophy by transcriptional and modular profiles. However, separation was not feasible, most likely because many of these patients have several of these conditions at the same time [15]. The majority of these CVID patients also over-expressed transcripts within erythrocyte-related modules as found in previous studies of subjects with autoimmune or infectious disease [22,32].

Focusing on dominant genes contributing to the differential transcriptional signatures distinguishing all CVID subjects from healthy controls, the most significantly unregulated genes were those contained within the IFN pathways, comprising 15 of the 20 most significantly up-regulated transcripts (Table 3). In fact, the signature for CVID subjects considered as a group, was quite dominated by the IFN profile contributed by CVID subjects with inflammatory complications. This became clear when CVID subjects without these complications were compared to healthy controls. Here the IFN signature disappeared, and several erythrocyte modules were noted (Table 4). Again displaying differences between these groups, when CVID subjects with and without inflammatory conditions were compared to each other, 16 of the 20 most significantly upregulated transcripts were identified as IFN related (Table 5). As expected, the most consistently down-regulated genes for CVID subjects in general, were B and T cell, plasma cell and immunoglobulin structural genes, not a surprising finding in this patient population with hypogammaglobulinemia and known adaptive immune defects (Table 6).

**Table 3:** Up-regulated genes: Healthy Controls vs. all CVID subjects.

**Gene**	**Name**	FC*	**Module**
HBD	Hemoglobin delta chain	12.56	Erythrocytes
SERPING1	Serpin peptidase inhibitor (C1 inhibitor)	9.16	IFN
IFI27	Interferon alpha-inducible protein 27	8.10	IFN
ANKRD22	Ankyrin repeat domain 22	5.86	
RSAD2	Radical S-adenosyl methionine domain containing 2	5.02	IFN
FCGR1B	Fc fragment of IgG, high affinity Iβ receptor	5.01	
EPSTI1	Epithelial stromal interaction 1	4.67	IFN
BATF2	Basic leucine zipper transcription factor	4.55	IFN
FCGR1A	Fc fragment of IgG, high affinity Iα receptor	4.31	
RAP1GAP	RAP1 GTPase activating protein	4.23	
IFI44L	Interferon-induced protein 44-like	4.09	IFN
IFIT3	Interferon-induced protein	3.99	IFN
GBP5	Guanylate binding protein 5	3.75	IFN
CXCL10	Chemokine (C-X-C motif) ligand 10	3.72	IFN
GBP6	Guanylate binding protein family member 6	3.59	IFN
GBP1	Guanylate binding protein 1, interferon-inducible	3.55	IFN
ETV7	Ets variant 7	3.43	IFN
ISG15	IS*G15* ubiquitin-like modifier	3.38	IFN
FBXO6	F-box protein 6	3.27	IFN
OAS3	2’-5’-oligoadenylate synthetase 3	3.14	IFN

* fold change.

**Table 4:** Up-regulated genes: Healthy Controls vs. CVID without inflammatory complications.

**Gene**	**Name**	FC*	**Module**
HBD	Hemoglobin subunit delta	7.02	Erythrocytes
EPB41	Erythrocyte membrane protein	2.62	Erythrocytes
C4BPA	Complement component 4 binding protein	2.42	
MXI1	MAX-interacting protein 1	2.33	Erythrocytes
KIF1B	Kinesin family member 1B	2.28	
PAK2	Serine/threonine-protein kinase	2.23	
PI3	PI 3-kinases	2.21	
STAU1	Double-stranded RNA-binding protein Staufen	2.21	
TCEA1	Transcription elongation factor A protein	2.19	
NAIP	NLR family, apoptosis inhibitory protein	2.18	
SOX6	Transcription factor SOX-6	2.18	
LILRA5	Leukocyte immunoglobulin-like receptor,	2.18	
FCGR1B	Fc gamma RI family	2.17	
CREBBP	CREB-binding protein	2.17	
OTX1	Homeobox protein OTX1	2.12	
BCLAF1	Bcl-2-associated transcription factor 1	2.12	
RORA	RAR-related orphan receptor A	2.06	
CLIP1	CAP-GLY domain containing linker protein 1	2.06	
TBC1D23	Innate immunity signaling	2.06	
FKBP3	FK506-binding protein 3	1.99	

* fold change.

**Table 5:** Up-regulated genes: CVID w complications vs. CVID w/o complications.

**Gene**	**Name**	FC*	**Module**
ANKRD22	Ankyrin repeat domain 2	3.83	
BATF2	Basic leucine zipper transcription factor,	3.85	IFN
CMPK2	Cytidine monophosphate (UMP-CMP) kinase 2	2.35	IFN
CXCL10	Chemokine (C-X-C motif) ligand 10	3.28	IFN
EPSTI1	Epithelial stromal interaction 1	3.50	IFN
ETV7	Ets variant 7	2.41	IFN
FBXO6	F-box protein 6	2.29	IFN
FCGR1A	Fc fragment of IgG, high affinity Iα, receptor	2.47	
FCGR1B	Fc fragment of IgG, high affinity Iβ	2.75	
GBP1	Guanylate binding protein 1	2.69	IFN
GBP5	Guanylate binding protein 5	2.93	IFN
GBP6	Guanylate binding protein 6	2.82	IFN
IFI27	Interferon, alpha-inducible protein 27	6.69	IFN
IFI35	Interferon-induced protein 35	2.30	IFN
IFI44	Interferon-induced protein 44	2.64	IFN
IFI44L	Interferon-induced protein 44-like	3.69	IFN
IFI6	Interferon, alpha-inducible protein 6	2.40	IFN
IFIT1	Interferon-induced protein with tetratricopeptide repeats 1	2.32	IFN
IFIT3	Interferon-induced protein with tetratricopeptide repeats 3	3.47	IFN
IGFL2	IGF-like family member 2	2.28	

* fold change.

**Table 6:** Down regulated genes: Healthy controls vs. All CVID.

**Gene**	**Name**	FC*	**Module**
IGJ	Immunoglobulin J polypeptide	-12.86	plasma cell
LRRN3	Leucine-rich repeat neuronal protein 3	-6.93	T cell
IGLL1	Immunoglobulin lambda-like polypeptide 1	-5.32	B cell
MYOM2	Myomesin-2	-4.96	
CNTNAP2	Contactin-associated protein-like 2	-4.87	B cell
FCGBP	Fc fragment of IgG binding protein	-4.43	
TNFRSF17	Tumor necrosis factor receptor superfamily member 17	-4.22	plasma cell
COBLL1	Cordon-bleu protein-like 1	-3.78	B cell
VPREB3	Pre-B lymphocyte protein 3	-3.55	B cell
TPM2	Tropomyosin beta chain	-3.53	
BACH2	Transcription regular protein BACH2	-3.52	
EPHA1	Ephrin receptor subfamily	-3.47	T cell
SLC16A10	Aromatic amino acid transporter	-3.16	T cell
COCH	Coagulation factor C	-3.11	
TAF15	TAF15 RNA polymerase II	-3.04	
OSBPL10	Oxysterol binding protein-like 10	-3.02	B cell
EFHC2	EF-hand domain (C-terminal) containing	-3.02	
TCEA3	Transcription elongation factor A	-2.81	T cell
AXIN2	Axin-like protein	-2.82	T cell
KLHL3	Kelch-like protein 3	-2.81	T cell

* fold change.

**Figure 9:**
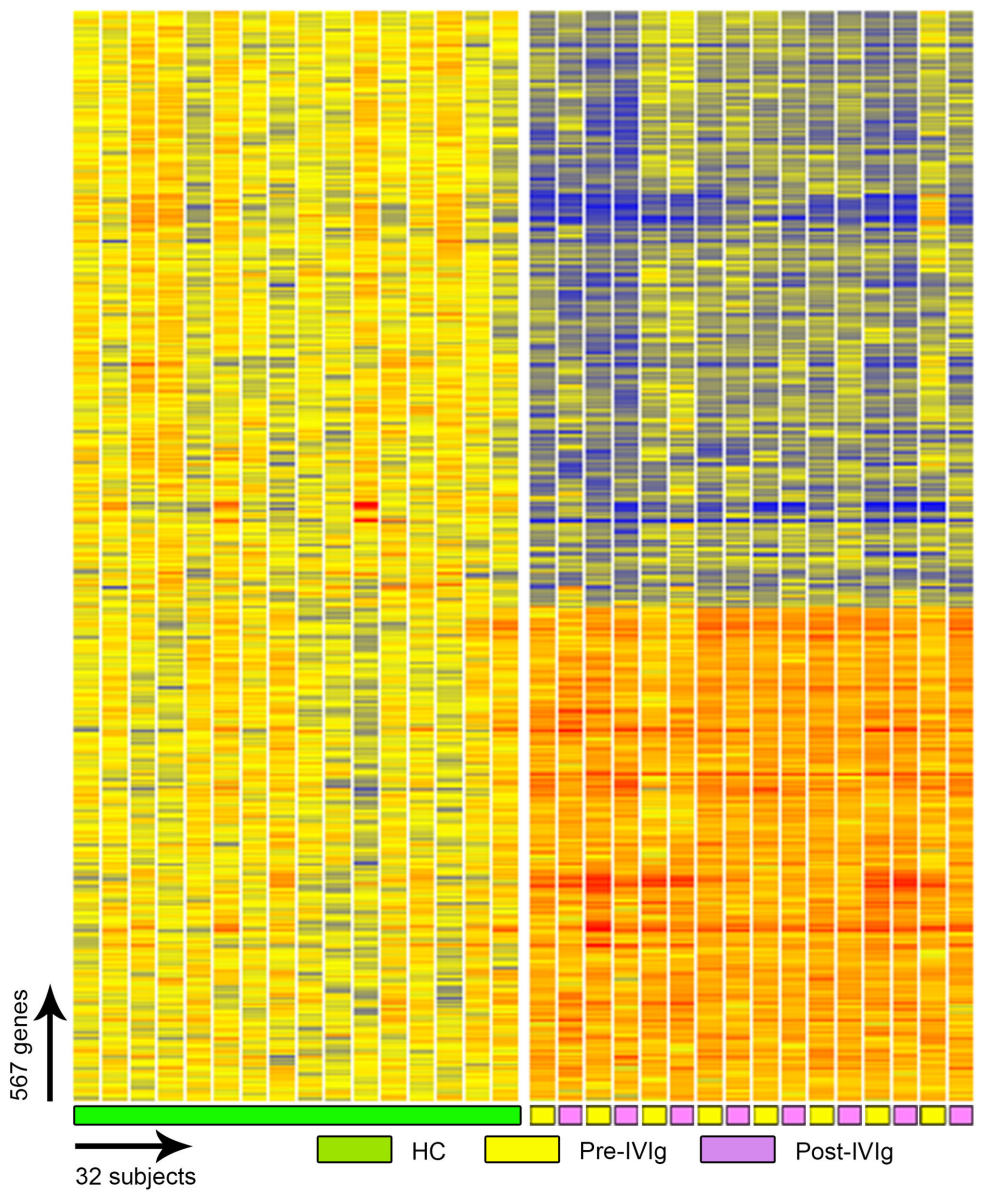
IVIg therapy and gene expression analysis. Eight additional CVID subjects were tested before and 5-7 days after receiving IVIg. Individual transcripts (567) were selected by Welch ANOVA test (MTC: Benjamini and Hochberg, FDR, p-cutoff <0.01) between healthy controls, pre-IVIg and post-IVIg patients.

Immune globulin therapy itself is known to exert a number of both up and down-regulatory effects on the immune system [33]. Thus to control for the potential effect of Ig therapy on the gene signatures of CVID patients, we sampled in a separate subgroup of patients before and 7 days after an IVIg infusion. The transcriptional patterns of these patients did not change for the most part, supporting that the IFN signature is not an effect of IVIG, nor altered by this therapy (Figure 9).

### Molecular basis of the signatures in CVID

**Figure 10:**
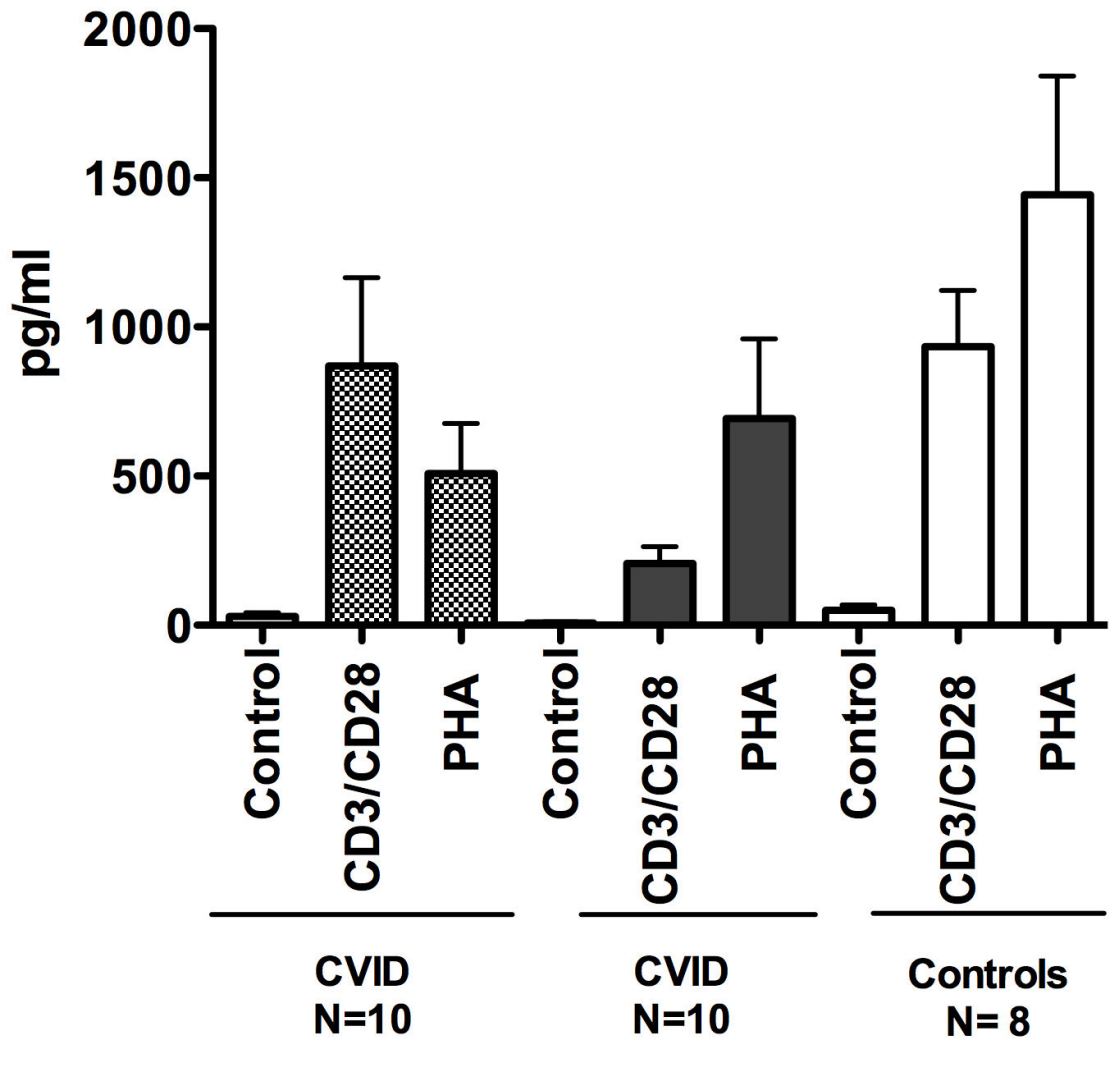
Production of IFN-γ. Culture supernatants of PBMCs of CVID subjects with (10) (CVID+) or without inflammatory (10) (CVID-) complications, activated with media alone, added CD3/CD28 beads or PHA, were assessed by ELISA for IFNγ production after 72 hours, and compared to supernatants of similarly treated cells from normal controls.

**Figure 11:**
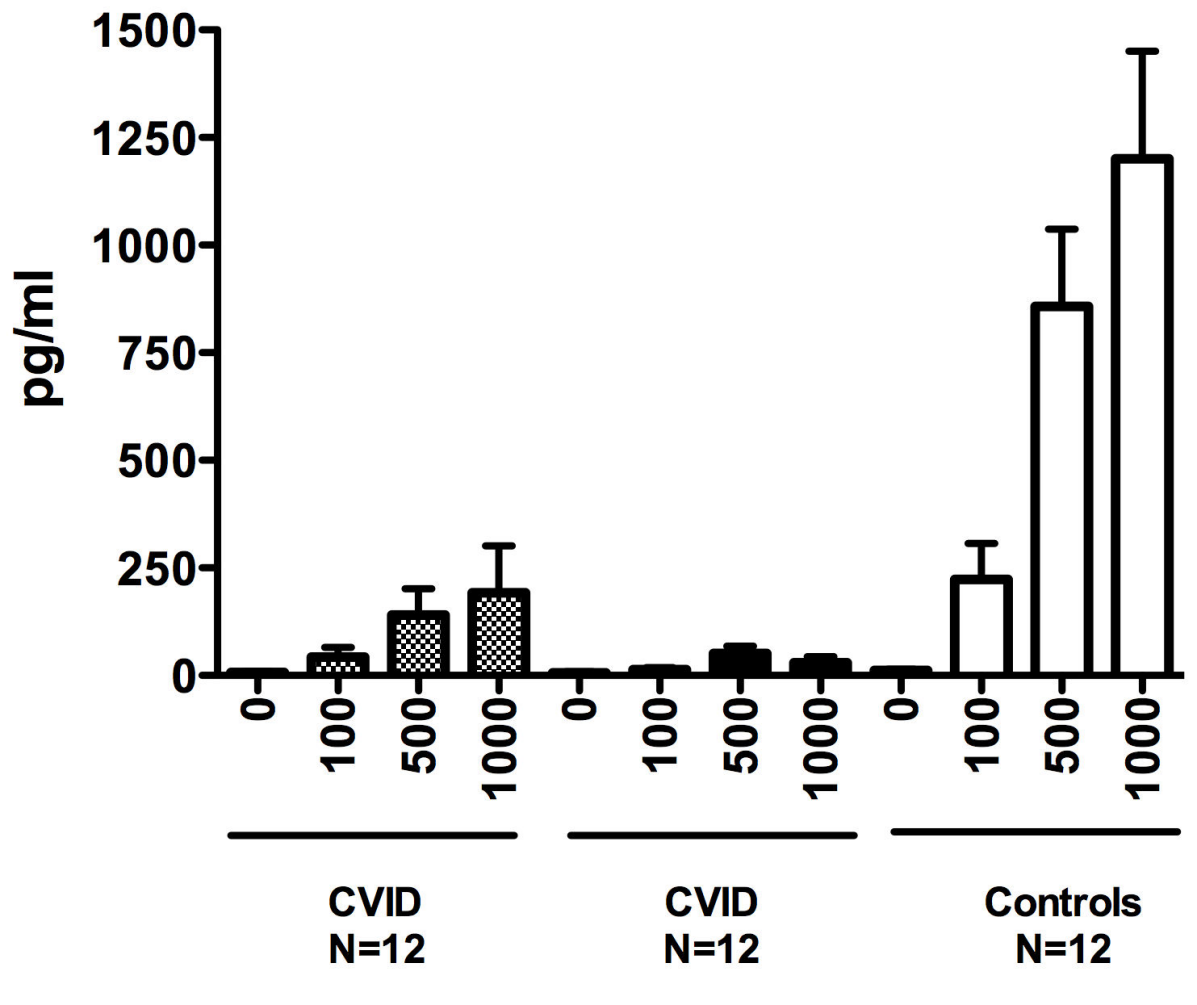
Production of IFN-α. For IFN-α production, PBMCs from control subjects (10) and CVID subjects with (+) and without inflammatory (-) complications, were stimulated with increasing amounts of loxoribine. IFN-α in supernatants was assessed by ELISA.

As the IFN signature was dominant for subjects with inflammatory conditions, we compared *in vitro* IFN production for subjects with and without these complications to healthy controls. However, supernatants of activated CVID PBMCs showed that for all conditions, CVID cells actually produced less IFN-γ than similarly treated control cells (for anti-CD3+ CD28, p= 0.03; for PHA p=0.02) and there were no differences between cells of CVID subjects with or without inflammatory complications (Figure 10). In addition, TLR7, TLR7/8 or TLR9 activated PBMCs (or as shown previously, this is also true for plasmacytoid dendritic cells) from CVID subjects produced less IFN-α than cells of normal controls [28,34]. However when segregated into subjects with and without inflammatory complications, mononuclear cells of subjects in the former group activated by TLR7 or TLR7/8 agonists produced significantly less IFN-α (p=0.003 and 0.005) than CVID subjects without these conditions (Figure 11). As plasmacytoid dendritic cells, the main IFN-α producers under these conditions were not enumerated, we are not able to discern if even fewer (or more impaired) cells in cultures of subjects with complications, might explain these differences.

## Discussion

The diagnosis of CVID is based on hypogammaglobulinemia with loss of production of functional antibody to vaccine or environmental antigens. Almost all subjects have a history of infections, but analyses of both European and US patient cohorts show that 40 to 50% of subjects have significant but poorly understood, apparently non-infectious inflammatory complications. These include organ-specific or hematologic autoimmunity, gastrointestinal inflammatory disease, marked lymphoid hyperplasia, usually with splenomegaly, and/or lymphocytic and granulomatous lymph tissue and organ infiltrations [15,24]. While Ig replacement is the standard therapy for all, those subjects suffering from inflammatory complications have both increased morbidity and worse survival [10,15]. The numbers and phenotypes of peripheral B cells provide some clues about clinical outcomes [35,36,37,38] [30,36,39,40] but these analyses have not elucidated the pathogenesis of these complications, and have not suggested therapeutic options.

This study provides the first analysis of whole blood transcriptional profiles of subjects with CVID, allowing subjects with and without inflammatory complications to be distinguished from each other and from healthy controls. The data suggest unique pathologic differences between these groups. Both CVID and XLA subjects have a characteristic loss of antibody production, receive Ig therapy and share similar down-regulated B cell pathways. However, CVID subjects display a unique signature, including a down-regulation of T cell-related transcripts not found in XLA, illustrating the intrinsic differences between these defects of humoral immunity. The up-regulation of a unique signature in CVID subjects with inflammatory disease, a signature not observed in XLA, is in line with the fact that subjects with XLA are largely exempt from the non-infectious complications found in CVID [41]. While Ig infusions are known to have a variety of immune modulating effects [33], the infusion of Ig, at least in the standard doses used in regular reconstitution used here, did not alter the inflammatory mRNA signature of the CVID subjects tested. In addition, as these subjects were already on maintenance Ig therapy at regular intervals, alteration from this baseline might not be expected.

One of the main findings of our study is the presence of an IFN signature, predominantly in CVID patients with inflammatory complications. An IFN signature is characteristic of an immune responses to viral vaccinations [42], as well as chronic viral diseases including HTLV-1 [20], hepatitis C [43] and HIV [44]. Subjects with CVID studied here, being immune deficient, may be susceptible to viral infections, but none who had symptomatic infections were enrolled. With some possible exceptions [45,46], chronic viral infections have also not been demonstrated in this syndrome, thus viral activation, while not eliminated as a possibility, does not appear to be a likely cause of the IFN signature observed. However, Type I and Type II IFNs are known mediators of chronic autoimmune diseases such as systemic lupus erythematosus (SLE), dermatomyositis, and Sjogren’s syndrome, and rheumatoid arthritis, etc [47] [48]. An INF signature has also been noted in the context of other chronic inflammatory conditions, some genetic [49,50] [51], and some induced by over expression of selected IFN gene pathway members [52]. In SLE, the chronic activation of the Type I IFN pathway might result from host DNA and RNA present within immune complexes (ICs) that could activate pDCs through endosomal TLRs. We did not observe an activation of the neutrophil module (Figure 3a, module 5.15), unlike pediatric SLE blood, where this cellular signature has been linked to IFN-α overproduction in this autoimmune disease [18,53]. An IFN signature (both IFN-αβ and γ) has also observed in active tuberculosis, which on treatment, resolved [19] [54]. In subjects with active tuberculosis, IFN-α2 and IFN-γ proteins were not elevated in serum although as for CVID subjects, IFN-inducible chemokine CXCL10 (IP10) and other IFN responsive genes, were significantly increased. Showing that this signature is not found in other infection models, patients with bacterial infections due to *Staphylococcal aureus*, did not show a prominent IFN signature even though transcripts from neutrophil pathways were increased [31,54]. As shown previously [28,34], Toll like receptor 7 or 9 activated PBMCs or plasmacytoid dendritic cells from CVID patients produced little IFN-α detectable by ELISA in culture. The production of IFN-γ by CVID patients PBMCs was also overall reduced when compared to control subjects. Impaired secretion of these cytokines in [55] culture would not exclude excess *in vivo* production as continued TLR activation leading to pathway activation, may desensitize the relevant cells leading to exhaustion [56].

In addition to the predominant IFN signature, blood of CVID patients with inflammatory disease also had over-expressed transcripts contained within erythrocyte-related modules. The significance of this over-expression is not clear. While greater degrees of anemia might provide a logical explanation, comparison of the cohorts did not reveal clear differences. However, we and others have described an erythropoiesis signature in patients with auto-inflammatory diseases and Gram (+) bacterial infections, both of whom also have strong IFN signatures. Investigating these connections, IL-6, IL-17, IL-10 and IL-1 cytokine activation has been demonstrated suggesting the activation of shared innate pathways in autoimmunity [22,32,57,58]. Other work has more directly connected interferon (IFN) signaling-related genes and STAT-family members in the transcriptional control of the erythroid network [59].

Other distinguishing features of CVID subjects with inflammatory conditions were more severe decrease in absolute lymphocyte counts, far fewer circulating B cells and isotype switched memory B cells. The blood transcriptome analysis revealed that these subjects also had a significant down-regulation of T cell, B cell and plasma cell modules. This indicates that CVID subjects with inflammatory conditions are likely to suffer from a more significant defect of adaptive immunity than those CVID subjects without inflammatory conditions. These data are in line with previous observations that CVID subjects with fewer isotype switched memory B cells, more impaired thymic and B cell bone marrow output, or reduced numbers and function of regulatory T cells, have a significantly greater likelihood of inflammatory consequences [29,30,60,61,62]. Greater losses of adaptive immunity could lead to an expansion of innate immune responses and potentially the global IFN signature observed. In these subjects, residual mRNA from still viable organisms, could provide an endogenous danger signal, initiating the recently described TIR-domain-containing adaptor protein inducing IFNβ (TRIF) dependent innate inflammatory pathway, leading to the production of large amounts of IFN [63]. Alternatively with poorer adaptive immunity, these subjects may have an activation of other inflammatory cell populations such as IFN producing innate lymphoid cells, not examined here [64]. However, regardless of factors leading to an intense IFN signature in CVID subjects with inflammatory conditions, the unbiased use of this whole blood transcriptome analyses may provide a tool for distinguishing CVID subjects who are at risk for increased morbidity and earlier mortality. As more effective therapeutic options are developed, whole blood transcriptome analyses could also provide an efficient means of monitoring the effects of treatment of subjects with this inflammatory phenotype.
